# Leading Emerging and Diverse Scientists to Success: Results from LEADS alumni

**DOI:** 10.1017/cts.2022.513

**Published:** 2023-02-10

**Authors:** Doris Rubio, Marie K. Norman, Colleen A. Mayowski, Chelsea N. Proulx, Alana Karras, Leslie Hausmann, LEADS Site Leaders Team

**Affiliations:** 1 Institute for Clinical Research Education, University of Pittsburgh School of Medicine, Pittsburgh, PA, USA; 2 Center for Health Equity Research and Promotion, Veterans Affairs, Pittsburgh Healthcare System, Pittsburgh, PA, USA

**Keywords:** Diversify the biomedical research workforce, Minority Serving Institutions, research training, career development

## Abstract

**Purpose:**

In 2015, the University of Pittsburgh partnered with several Minority Serving Institutions to develop the Leading Emerging and Diverse Scientists to Success (LEADS) Program. LEADS was designed to provide skills development, mentoring, and networking support to early career underrepresented faculty.

**Method:**

LEADS included three components: skills training (e.g., grant and manuscript writing and team science), mentoring, and networking opportunities. Scholars completed a pre- and post-test survey and an annual alumni survey that included measures on burnout, motivation, leadership, professionalism, mentoring, job and career satisfaction, networking, and an assessment of their research self-efficacy.

**Results:**

Scholars demonstrated a significant increase in their research self-efficacy having completed all the modules (*t* = 6.12; *P* < 0.001). Collectively, LEADS scholars submitted 73 grants and secured 46 grants for a 63% success rate. Most scholars either agreed or strongly agreed that their mentor was effective in helping to develop their research skills (65%) and provided effective counseling (56%). Scholars did experience increased burnout with 50% feeling burned out at the exit survey (t = 1.42; *P* = 0.16) and 58% reporting feelings of burnout at the most recent survey in 2020 (t = 3.96; *P* < 0.001).

**Conclusions:**

Our findings support the claim that participation in LEADS enhanced critical research skills, provided networking and mentoring opportunities, and contributed to research productivity for scientists from underrepresented backgrounds.

## Introduction

To produce the most rigorous, nuanced, and impactful scientific investigations of contemporary healthcare issues [1], the biomedical research workforce urgently needs to recruit and retain scholars from diverse backgrounds [2–6]. Underrepresented researchers bring new and important perspectives to bear on critical research questions, as well as access to and insight into racially, ethnically, and socioeconomically diverse patient populations [7].

Yet the number of underrepresented biomedical researchers, particularly those who identify as Black, Latinx, and Native American, remains alarmingly low [1,8–10]. Even when researchers from underrepresented groups pursue biomedical research careers, there continues to be significant attrition [11] and lower rates of promotion than among their non-Latinx White counterparts [12]. According to a 2021 report from the National Science Foundation, of faculty positions in scientific research in 2019, only 3% were held by Black researchers, 4.7% by Latinx researchers, and 0.2% by Native American researchers [13].

At the same time, several factors have been identified that positively affect underrepresented faculty persistence in biomedical research. These include 1) skills development [14–16]; 2) mentoring [8,17–20]; and 3) opportunities for networking [14]. In particular, programs that intervene at the assistant professor (early career) level have been shown to increase representation and prevent attrition among underrepresented faculty [21].

In 2015, the University of Pittsburgh partnered with several Minority Serving Institutions (MSIs) to develop the Leading Emerging and Diverse Scientists to Success (LEADS) Program to provide research skills training, mentoring, and networking support for underrepresented early career investigators working at partnering MSIs. A previous article [9] describes the process by which LEADS was developed. This paper presents outcomes for the first four cohorts of participants in the LEADS Program, with success of the program measured by changes in research self-efficacy along with number of grants and publications.

## Methods

LEADS is a 1-year online training program for early career investigators – faculty and postdoctoral trainees – at participating MSIs. The program was initially developed as a collaboration between University of Pittsburgh and Charles Drew University, Morehouse University, University of Hawaii Manoa, and Universidad De Puerto Rico Medical Sciences Campus. From 2015 to the present, the number of MSIs participating in LEADS grew from four to nine out of an increase in national interest and an effort to extend to reach of LEADS. The additional MSIs included Hampton University, Howard University, Meharry Medical College, North Carolina Central University, and the University of Texas, San Antonio. These partnerships were formed based on word of mouth. Each site had a senior leader who helped recruit participants and served as a mentor when needed.

### Participants

We recruited postdoctoral fellows and early career faculty from the participating MSIs. Each site had a designated site leader who helped with recruitment by distributing advertisements to their institution’s faculty and serving as a contact point for interested applicants. We encouraged all interested applicants to discuss participation in LEADS with their site lead, for both application questions and also for first-hand insight into the LEADS Program [9]. We also advertised by emailing promotional material across the Research Centers in Minority Institutions (RCMI), which is composed of 18 MSIs and includes most of the MSIs participating in LEADS. We also relied on word of mouth from previous LEADS scholars and sent our promotional material to all LEADS alumni to distribute across their networks.

We collected applications from January to March. The LEADS application, which was typically due April 1, required two statements from the applicant describing (1) why they were interested in LEADS and (2) their future career goals. Applicants were also required to include their CVs and attach a letter from their department chair, indicating that the applicant would have 20% protected time to participate in LEADS. The LEADS application was specifically made to be a low burden; however, we did note that many applicants were not able to secure a letter indicating that they had protected time, either because the chair would not provide it or because the applicant was reluctant to ask. Because we did not want to exclude those applicants, we did not use protected time as a review criterion.

Applications were reviewed by the ICRE Diversity Committee, a diverse group of faculty and administrators from across the health sciences schools at the University of Pittsburgh. Participants were selected based on three criteria: they needed to (1) be affiliated with the MSI, (2) be pursuing or planning to pursue a research career, and (3) have the support of a mentor at their institution. No specific emphasis was put on ensuring a gender or racial/ethnic balance among participants.

### Program Components

In keeping with the factors previously mentioned that positively affect underrepresented faculty persistence in biomedical research, LEADS involved three basic components: skills training, mentoring, and networking opportunities. These are explained at length in a previous paper [9] and are briefly described below. We purposefully designed the components of LEADS to be as flexible as possible, to avoid adding burden to already over-extended participants. So, while all the components were required, we allowed flexibility as to when things were completed. We initially anticipated 8–10 hours per week to be devoted to participation in the LEADS (equating to 20% protected time). However, it quickly became apparent that scholars could only give about 3–5 hours per week. So, we adjusted the workload accordingly.

1. *Skills Training*: LEADS offered participants intensive, instructor-led, online modules targeting key research topics. Modules ranged from 2 to 8 weeks long (most were 4 weeks) and focused on the development of skills that are essential for professional success but are not always included in the formal curriculum of biomedical research training programs. These included topics such as grant writing, medical writing, team science, and critical/creative thinking (see Table 1). For each module (one unit = one week of a module), scholars engaged asynchronously with relevant videos and readings, discussion boards, and assignments. Many of the activities provided scholars an opportunity to get feedback on their own work products, such as a grant or manuscript. Each unit also included one, online, synchronous session for 1.5 hours. These sessions prioritized conversation among scholars – thus serving as a networking opportunity but also a safe space for scholars to ask questions about how the topics in the unit related to their own work and career trajectory. Completion of these modules was a required component of LEADS. Those scholars who were unable to complete the modules were given the opportunity to make up the work through the completion of relevant work products.
  The instructors for the modules were faculty at the University of Pittsburgh and University of Puerto Rico. Prior to offering the program, the team of instructors met biweekly with an online education expert. The expert developed templates for us to create the modules for consistency. She also worked individually with each instructor guiding them on the development of the modules. We arranged the modules in a very specific order so that the scholars would be poised to launch their research careers.
2. *Mentoring*: To ensure that LEADS scholars received mentoring, LEADS also established agreements with the senior leaders at participants’ home institutions to provide mentoring. The program also offered career coaching training to these senior leaders to further expand their mentoring toolkit. The career coaching techniques taught differed from and complemented traditional mentoring by using active listening and powerful questions to help mentees identify their own passions and author their own careers. Institutional leaders agreed to meet monthly with LEADS scholars from their institution. It is important to note that the senior leaders were not the research mentors for the scholars. We did not connect with any of the research mentors.
3. *Networking*: To foster the type of networking that leads to productive collaborations, LEADS included components to cultivate community and build connections among geographically distributed participants. Before the modules even began, we held an online orientation kick-off meeting. We reviewed the expectations of the programs, introduced Moodle (online Content Management System), and blocked time for scholars to introduce themselves and get to know one another. Scholars also had the opportunity to interact with the weekly synchronous sessions and the use of discussion forums in every online module. We aimed to foster a sense of community, facilitate networking, and promote connection across institutions and geographical regions.

**Table 1.** LEADS modules titles

Module name	Description of modules
Introduction to team science	This module covers the basics of team science. We discuss how to bring together the right mix of collaborators to enhance the success and impact of your research. We also review best practices for building and maintaining positive and productive working relationships with diverse collaborators from different disciplines, professions, and social backgrounds
Critical and creative thinking	The purpose of this module is to get scholars to think critically and creatively about their research. The module has discussion and exercises focused on how to make their research more innovative
Identifying the problem	In this module, scholars learn how to define a significant research problem, articulate important, researchable questions, and use a conceptual framework to develop testable hypotheses based on your questions
Asking the right question	This module discussed criteria to evaluate the significance of a problem and prioritize questions. By the end, scholars should be able to develop questions to guide the investigation of an impactful problem
Grant writing	In the grant writing I and II modules, scholars learn critical elements of the grant preparation and writing process, develop a specific aims page and extended research strategy outline, and provide constructive feedback to other participants in the modules
Starting your research	This module is designed to help scholars get their research started. Topics such as IRB, operations manual, and even hiring and firing are discussed
Medical writing and communication	This module instructs scholars to present their work clearly and effectively in many written forms to successfully advance their careers. Scholars learn how to write effectively composed abstracts and peer-reviewed manuscripts
Effective peer reviewing	In this module, scholars learn how to do a scientific peer review. They are instructed on how to evaluate scholarly work by colleagues to enhance the quality of scholarship. This module is designed to de-mystify many key aspects of peer review
Launching your research career	This module focuses on self-discovery. Several experiential exercises are designed to help scholars develop strategies for a successful research career. At the end of the module, scholars have a 5-year plan

Networking was further facilitated by the addition, in 2018 (year two of the grant), of a 3-day LEADS Summit, featuring keynote speakers, training workshops, mentoring, networking, and opportunities to receive feedback on works in progress. We have since offered the Summit annually at a participating MSI – except for 2021–2, which, due to the pandemic, were held virtually. While attending the LEADS Summit is not a requirement of the fellowship, current LEADS scholars and alumni are welcomed at the Summit to renew acquaintances, foster new connections, and receive further career training.

Because scholars expressed that the connections they made through the program were impactful and that they would be interested in additional networking activities, we offered optional works in progress calls, where alumni and program leaders met monthly to discuss their research progress. To spur research productivity and to provide additional guidance on grant and manuscript writing, we also periodically offered optional grant writing and manuscript writing sprints.

### Data Collection

LEADS scholars completed a pre-test survey and a post-test survey at the conclusion of the first year of the program. Alumni received a survey every year after they completed the program, with the most last survey in 2020 being included in these analyses. We decided to not include the 2021 cohort as we were concerned about the impact that the pandemic had on their participation in LEADS. Many of the instructors expressed concern about the significant decline in participation compared to previous years.

The survey was identical across time points, with the exception of the post-test survey, which included additional items related to program satisfaction. Each survey included measures on research self-efficacy, burnout, motivation, leadership, professionalism, mentoring, job and career satisfaction, and networking. For these measures, we used previously developed measures and modified to best meet our purpose. For example, we used the Clinical Research Appraisal Inventory and modified it to best align to the objectives of the modules.

This study was deemed not human subjects research by the University of Pittsburgh Institutional Review Board.

### Analyses

We used Stata Version 14.2 (College Station, TX; StataCorp LLC) for all analyses. Paired t-tests were used to analyze changes in average scores from pre-test to post-test survey and from pre-test to the most recent survey (post-test or alumni) for all survey measures for the first four cohorts.

## Results

Since 2016, LEADS has enrolled five cohorts of scientists (we opted to not use the last cohort due to the impact that the pandemic had on their engagement with LEADS), with a total of 67 trainees from 12 different institutions (see Table 2). Most participants (87%) were from an underrepresented background as defined by NIH [22] (identified as African American, Hispanic, or Disadvantaged) and were female (81%). Most of the scholars had a PhD or equivalent (e.g., EdD (72%)). Additional participant characteristics are described in Table 2. The University of Puerto Rico had the highest number of LEADS scholars (N = 14; 21%). Cohort response rates from pre-test to post-test ranged from 71 to 85% and 82 to 92% from pre-test to most recent.

**Table 2.** Demographic characteristics of LEADS scholars

	*n*	%
Total scholars	67	
Gender		
Female	54	81
Male	12	18
Prefer not to answer	1	1
Underrepresented backgrounds	58	87
African American	36	54
Hispanic	20	30
Disadvantaged	17	25
Degrees		
Professional clinical degrees (e.g., MD, PharmD)	11	16
PhD or equiv.	48	72
Professional degree and PhD	8	12
Minority-serving institution		
Charles Drew	1	1
Hampton	1	1
Howard	7	10
Meharry	11	16
Morehouse	6	9
University of Hawaii	12	18
University of Puerto Rico	14	21
University of Texas, San Antonio	4	6
North Carolina Central University	11	16
Cohort		
2016	12	18
2017	13	19
2018	17	25
2019	11	16
2020	14	21

### Outcomes

#### Research skill inventory

We created a measure to assess scholars’ confidence in their research skills before and after the program. We developed the research skills self-efficacy measure (19 items) to specifically measure skills that LEADS modules emphasized, such as *identify a funding agency, submit a competitive grant*, and *respond to reviewers’ critiques.* We averaged scholar responses across each research skills efficacy item to create a global score, given any one research skill may not lead to research success. Reliability (internal consistency) for the measure was strong at baseline (Cronbach’s α = 0.94). Item-wise comparisons were also made for the research skills efficacy measure to understand which skills improved, and therefore, which modules may have had an initial or sustained impact on skill building.

We found a significant improvement in scholars’ overall confidence in their research skills once they completed all of the modules (t = 6.12; *P* < 0.001), which was maintained at the most recent survey (t = 5.05; *P* < 0.001) (see Table 3). In fact, of the 19 items, all of them increased from pre to post. The skills that showed the greatest improvement were *develop a research question using FINER criteria* (t = 7.44; *P* < 0.001), *identify the stages of the grant writing and review process* (t = 6.27; *P* < 0.001), and *identify appropriate funding agencies for your research* (t = 5.21; *P* < 0.001). This difference was sustained in the most recent survey (t = 5.05; *P* < 0.001). And as we found from pre-test to post-test, *develop a research question using FINER criteria* maintained the highest improvement (t = 5.78; *P* < 0.001) followed by i*dentify appropriate funding agencies for your research* (t = 4.83; *P* < 0.001) and *submit a competitive grant application* (t = 4.61; *P* < 0.001).

**Table 3.** Research skills inventory: comparison between three time points^+^

Please indicate your ability to successfully perform each task by selecting a single number from 0 to 10 that best describes your level of confidence. We would like to know how confident you are that you can successfully perform these tasks TODAY	Pre-test		Post-test^#^		Most recent^^^	
	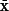	sd	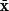	sd	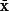	sd
Average of all items	6.84	1.14	7.46^*^	1.73	7.83^*^	1.09
Assemble an interdisciplinary, transdisciplinary, or multidisciplinary research team	6.98	1.96	7.46	1.73	7.33	1.96
Manage an interdisciplinary, transdisciplinary, or multidisciplinary research team	6.46	1.84	7.02	1.96	6.96	2.05
Critically evaluate arguments	7.29	2.08	7.83	1.01	8.00	1.14
Approach research problems creatively	7.69	1.88	8.02	1.26	7.70	1.23
Identify a significant research problem	7.40	2.14	8.02	1.54	7.85	1.48
Launch a research project	7.21	1.88	7.88	1.78	7.50	1.53
Distinguish between a theoretical and conceptual model	6.49	2.39	7.83^*^	1.56	7.78^*^	1.46
Develop a research question using FINER criteria	4.67	3.12	8.12^*^	1.40	7.72^*^	1.61
Develop a hypothesis based on your research question	7.34	2.15	8.61^*^	1.59	8.22	1.51
Identify the sections of a scientific research paper	8.10	2.16	8.95	1.78	8.98	1.63
Write a compelling abstract for a scientific paper	7.32	2.03	8.46^*^	1.45	8.44^*^	1.37
Write a scientific research paper	7.05	2.43	8.35^*^	1.75	8.05	1.77
Identify the stages of the grant writing and review process	6.63	2.48	8.43^*^	1.77	7.95^*^	1.70
Identify appropriate funding agencies for your research	6.10	2.77	7.81^*^	2.24	7.43^*^	2.03
Submit a competitive grant application	5.46	2.87	7.15^*^	2.12	6.87^*^	2.40
Respond to reviewer critiques of a manuscript	6.78	2.82	8.10^*^	1.77	8.14^*^	1.68
Describe the roles and responsibilities of a peer reviewer	7.18	2.39	8.63^*^	1.73	8.49^*^	1.73
Develop a timeline for completing a research project	7.14	1.77	8.26^*^	1.82	7.85	1.81
Write a manual of procedures (or an equivalent document) for a research project	6.88	2.35	7.93	1.88	7.60	1.92

+ Only tested those items where the difference was > 1 point improvement to minimize type I error.

# Pre-test vs post-test after completing the first year of the program using paired t-test.

^ Pre vs most recent (post-test or alumni survey) using paired t-test.

* Significant using Bonferroni-corrected alpha.

Two of the modules focused on grant and manuscript writing. Overall, the LEADS scholars collectively published 132 articles, submitted 73 grants, and secured 46 grants for a 63% success rate. Of the 73 grants that were submitted, 51 (70%) were either federal funding or from a foundation (Table 4).

**Table 4.** Grants submitted and funded by scholars

Funding	Grants submitted	Grants funded	Success rate
Federal	30	16	53%
Institutional	14	8	57%
Supplement	6	5	83%
Foundation	15	11	73%
Pilot	6	4	67%
Contract	2	2	100%

#### Mentoring

Most scholars (82%) reported having a primary mentor and/or mentoring team at baseline. Of those who had mentors, they had been working with their mentor(s) for about 3 years prior to LEADS and reported meeting with their mentors approximately twice a month. Most scholars either agreed or strongly agreed that their mentor was effective in helping to develop their research skills (65%) and provided effective counseling about career development and balancing professional responsibilities (56%). Only 41% thought their mentors were effective in counseling about balancing professional and personal life. None of the responses improved from pre- to post-test or pre-test to the most recent survey (*P* ≥ 0.05).

#### Increased levels of burnout

We did see some increase in burnout among the scholars over the years with 33% reporting feelings of burnout at baseline, compared to 50% feeling burned out at the exit survey (t = 1.42; *P* = 0.16) and 58% reporting feelings of burnout at the most recent survey in 2020 (t = 3.96; *P* < 0.001).

## Discussion

We found that participation in LEADS increased LEADS scholars’ confidence in their research skills; furthermore, scholars were extremely productive in securing grants and publishing their research. Scholars achieved a 63% grant funding rate. According to two professional organizations, the average acceptance rate for grants is 10–30% [23,24]. They are even farther above the application success rate for NIH research project grants (20.6% in 2020) [25]. Of note, success rates for NIH grants led by underrepresented researchers are 10 percentage points lower than grants led by White researchers [26]. Moreover, data repeatedly show that White scientists funding rate is 1.7-fold higher than for African American/Black scientists [27]. Scholars’ 63% success rate with grant funding is remarkable by any standards.

Likewise, LEADS scholars’ productivity as measured through submitted publications is evidence of the program’s impact. Collectively, scholars published 132 articles in a wide variety of academic journals during and after their participation. Although we lack a comparison group as discussed in the “Limitations” section below, anecdotally, many scholars have told us that they would never have reached this level of productivity had it not been for LEADS instruction and support. For example, one of the scholars got a Fulbright award and he stated, “Would NOT have been able to put together the proposal I did without all of the training and support I’ve gotten through LEADS!!!!”

We tracked the LEADS scholars over time and regularly surveyed them to assess confidence in their research skills. Many of their research skills gained in the program persisted such as identifying an appropriate funding agency for their research as well as submitting a competitive grant application. These results are encouraging as they demonstrate that even after obtaining one’s terminal degree such as a PhD or MD, additional training can help in successfully competing for grants as well as publishing manuscripts.

Ginther *et al*. found in 2016 that women are less likely to submit grant applications [28]. We hypothesize that additional training, such as that offered by LEADS, may increase women’s confidence in their research skills, translating into an increase in grant submission and funding, thus laying the foundation for a successful research career. Most LEADS scholars were women (81%), which speaks to the success that the women who participated in LEADS achieved. As one woman said, she would never have negotiated for her academic position, and another said she never would have published her manuscript if it was not for LEADS.

The increase in burnout was not a surprising finding. Even as early as 2008, we found that women and underrepresented researchers are more likely to be burned out than males and other races, respectively [29]. Given most of our participants are women and also underrepresented, we would expect this demographic to have the highest levels of burnout. However, burnout levels of 58% of the participants are concerning. Anecdotally, our scholars have told us that more and more is expected of them and they already have high teaching loads or other responsibilities. And since the pandemic, many institutions have reduced resources, which puts an even bigger burden on faculty. In the next phase of LEADS, we will be focusing on burnout and working on ways to reduce it among our scholars. Certainly, more interventions like LEADS are needed to support junior faculty as they strive to launch their research careers.

These findings, taken together, support our claim that participation in the LEADS Program enhances confidence in critical research skills, provides networking and mentoring opportunities, and contributes to research productivity for scientists from underrepresented backgrounds.

The LEADS grant was renewed for another 5 years. So, we continue to recruit scholars from participating MSIs and across the RCMI network and offer the curriculum. We did extend the fellowship to 2 years with the first-year focus on the modules and the second year dedicated to skill sprints where scholars work on their own manuscript and grant proposals. Our sustainability plan is to work with the MSIs so that each institution can adopt a component of LEADS and continue to offer it beyond the life of the grant.

### Limitations

These results are very encouraging; however, some limitations should be noted. The biggest limitation is the lack of a comparison group. To investigate how LEADS scholars’ productivity compared to a non-LEADS cohort at the participating MSIs, we attempted to do a matched comparison cohort study, asking LEADS scholars to suggest a colleague at the same career stage who was comparable in every way except for completing LEADS. Our intention was to use the matched cohort as a comparison group. However, despite offering incentives, we were unable to recruit enough participants for the comparison group to conduct the study. It is worth mentioning that the challenges we encountered in simply finding a comparison cohort are indicative of the very problem that LEADS is working to rectify (too few scientists from underrepresented backgrounds in the biomedical workforce), which perhaps speaks to the importance of programs like it. We are also cognizant that LEADS scholars may possess a higher degree of self-motivation than non-LEADS colleagues, which may contribute toward their productivity. But this is always the case with voluntary training programs, because one never knows their motivation to pursue such training and if it varies from others who do not pursue. Despite not having a comparison group, we were able to compare our results to two professional organizations who noted that the average success rate was only 10–30% [23,24], as well as comparing this rate to NIH’s success rate of approximately 20%. Moreover, several papers have documented significantly lower success rates for those who are underrepresented, which makes the scholars’ success rate even more impressive.

Another limitation to this work occurred because of the iterative feedback we sought from scholars and site leaders. As a result of our responsiveness, LEADS has been slightly modified each year with new opportunities for the scholars (e.g., annual summits, grant and manuscript writing sprints). This makes it difficult to pinpoint exactly which elements of the program were the most beneficial. So, while we do not know if scholars’ success is a result of the modules or the other components that were added, we do know that scholars reported higher research self-efficacy following the modules. It should also be noted that multicomponent interventions are needed to address systemic biases in academic institutions and medical research, thus making it unlikely that any one component of LEADS would have a sustained impact on its own.

## Conclusion

LEADS is a longitudinal, multi-component program focused on skill building, mentoring, and networking. Early career faculty at MSIs who participated in the program reports higher research confidence and high levels of scientific productivity in terms of publications and grants.
